# MouseNet v2: a database of gene networks for studying the laboratory mouse and eight other model vertebrates

**DOI:** 10.1093/nar/gkv1155

**Published:** 2015-11-02

**Authors:** Eiru Kim, Sohyun Hwang, Hyojin Kim, Hongseok Shim, Byunghee Kang, Sunmo Yang, Jae Ho Shim, Seung Yeon Shin, Edward M. Marcotte, Insuk Lee

**Affiliations:** 1 Department of Biotechnology, College of Life Science and Biotechnology, Yonsei University, Seoul, Korea; 2 Center for Systems and Synthetic Biology, Institute for Cellular and Molecular Biology, University of Texas at Austin, TX 78712, USA

## Abstract

Laboratory mouse, *Mus musculus*, is one of the most important animal tools in biomedical research. Functional characterization of the mouse genes, hence, has been a long-standing goal in mammalian and human genetics. Although large-scale knockout phenotyping is under progress by international collaborative efforts, a large portion of mouse genome is still poorly characterized for cellular functions and associations with disease phenotypes. A genome-scale functional network of mouse genes, MouseNet, was previously developed in context of MouseFunc competition, which allowed only limited input data for network inferences. Here, we present an improved mouse co-functional network, MouseNet v2 (available at http://www.inetbio.org/mousenet), which covers 17 714 genes (>88% of coding genome) with 788 080 links, along with a companion web server for network-assisted functional hypothesis generation. The network database has been substantially improved by large expansion of genomics data. For example, MouseNet v2 database contains 183 co-expression networks inferred from 8154 public microarray samples. We demonstrated that MouseNet v2 is predictive for mammalian phenotypes as well as human diseases, which suggests its usefulness in discovery of novel disease genes and dissection of disease pathways. Furthermore, MouseNet v2 database provides functional networks for eight other vertebrate models used in various research fields.

## INTRODUCTION

Geneticists have achieved impressive progress in discovering disease-associated genes and genotypes directly in humans, but the functional validation and mechanistic follow-up studies of these genes typically relies heavily on the use of laboratory animals. The laboratory mouse (*Mus musculus*) is the experimental tool of choice for many biomedical researchers, as for example in immunology, cancer biology, and stem cell biology, and there are many ongoing efforts to characterize mouse biology. In spite of these extensive efforts, as of this study, many mouse genes remain un-annotated. For example, only 7872 mouse genes are annotated with Gene Ontology biological process (GOBP) terms (1) by direct experimental or literature evidence. Even when considering computationally inferred annotations, 4869 genes have no GOBP functional annotations at all. Thus, the assignment of functions to mouse genes is a major ongoing challenge.

One major approach to systematically identify gene functions is through the use of large-scale functional gene networks. A genome-scale functional gene network for the laboratory mouse, dubbed MouseNet, was previously constructed by Bayesian statistical integration of heterogeneous omics-data in the context of the international MouseFunc competition (2). MouseNet construction, however, was limited to data made available through the MouseFunc competition (3), which restricted the predictive power of MouseNet relative to the wealth of available mRNA expression and protein–protein interaction data now available. For example, as of September 2015, at least 80 000 mouse mRNA expression profiles measured by microarray or next generation sequencing (NGS) are freely available from the Gene Expression Omnibus (GEO) database (4), whereas fewer than 250 expression experiments were used for MouseNet. Thus, we anticipated that incorporating a large amount of the public genomics data will substantially improve the functional network of mouse genes.

Here, we present MouseNet v2 (http://www.inetbio.org/mousenet/), which represents a substantial improvement over the previous version in both performance and usability. By incorporating new large-scale experimental data including 8154 microarray samples selected from a total of 76 002 tested samples of GEO (4) and improved network inference algorithms, we observed significant improvements to accuracy as well as genome coverage by MouseNet v2, which now covers 17 714 mouse genes (>88% of coding genome, increased from 72% in v1). In addition to providing functional associations between mouse genes, MouseNet v2 serves as a platform for researchers to generate new functional hypotheses using the principle of guilt-by-association. The implemented network-assisted search algorithms can prioritize mouse genes for a pathway or a trait, and can prioritize functional concepts for a query gene that needs to be characterized. Therefore, MouseNet v2 is not only a database but also a hypothesis generation server.

Network edge information for the integrated MouseNet v2 as well as individual component networks are freely downloadable. These component networks can be used to test novel data integration methods and generate alternative versions of mouse gene networks. Moreover, a total of 183 co-expression networks inferred from 8154 microarray experiments in the GEO database are also available from the MouseNet v2 database. Given that GEO database provides information about study design and relevant biological context for the source expression data, co-expression networks of MouseNet v2 provide a useful resource for context-specific network analysis.

Other model vertebrates are also widely used in various fields of research. For this reason, the Mouse Genome Informatics (MGI) database (5) provides mouse orthologs for eight other vertebrates that contain more than 12 000 mouse orthologs to aid the transfer of functional information from mouse to other vertebrates: rat (*Rattus norvegicus*), chimpanzee (*Pan troglodytes*), Rhesus macaque (*Rhesus macaque*), dog (*Canis lupus familiaris*), cattle (*Bos taurus*), chicken (*Gallus gallus domesticus*), western clawed frog (*Xenopus tropicalis*), zebrafish (*Danio rerio*). MouseNet v2 provides gene networks transferred from mouse based on orthology, and allows network-search and hypothesis generation for these vertebrates.

## CONSTRUCTION

MouseNet v2 was constructed as previously described for other animal gene networks (6,7) with some modifications, as detailed in full in the Supplementary Online Methods. Comparisons with the previous MouseNet in terms of data sources and network inference algorithms are also summarized in Supplementary Table S1. MouseNet v2 is based on gene annotation from the NCBI Concensus CDS project (8) (GRCm38.p2, version 16 as of 17 April 2014). To learn functional associations between mouse genes, we generated a set of positive gold-standard gene pairs that share functional annotations according to GOBP (1) (downloaded on 3 March 2015) or the MetaCyc database (9) (downloaded on17 March 2015). To generate an accurate gold-standard data set, we only consider GO annotations supported by reliable evidence codes, such as IDA (inferred from direct assay), IMP (inferred from mutant phenotype), IPI (inferred from protein interaction), and TAS (traceable author statement). Functional couplings between mouse genes were inferred from five main data sources: mRNA co-expression across experimental conditions, genomic context similarity based on phylogenetic profiles (10) and gene neighborhoods (11), physical protein–protein interactions, and functional gene–gene associations transferred from other organisms by orthology relationships (associalogs) (12).

In order to infer functional links from mRNA co-expression patterns, we first evaluated the available sets of GEO microarray experiments (GSE), selecting only those sets that contained at least 12 microarray experiments and measuring whether or not those genes with highly correlated mRNA abundances across the set of microarray experiments also showed an increased tendency to share gold standard positive functional annotations. This filter removed a majority of microarray datasets from further analysis. In total, we tested 76 002 microarray samples, and ultimately inferred co-expression links from a subset of 183 GSE comprising 8154 microarray experiments. Each of the 183 co-expression networks were then integrated into a single co-expression network. Functional links based on genomic context methods were obtained by analyzing gene neighborhood in 1748 prokaryotic genomes and by analyzing phylogenetic profiles across 396 eukaryotic genomes. Literature-curated protein-protein interactions were obtained from iRefIndex v14.0 (13). Furthermore, we transferred associalogs from functional networks for human, fly, and yeast via orthology to mouse genes. Finally, we then integrated the 13 data-type specific mouse gene networks using the previously described weighted sum log-likelihood scoring scheme (14). The resulting functional network of mouse genes contains 788 080 co-functional links and covers 17 714 genes (>88% of mouse coding genome), which is substantially expanded over the coverage (72%) of MouseNet v1. The integrated MouseNet v2 and individual component networks are summarized in Table 1. MouseNet v2 and all component networks, including 183 co-expression networks, are available from the network download page of www.inetbio.org/mousenet/.

**Table 1.** MouseNet v2 and component networks inferred from 13 distinct data types

Network	Description	Genes	Links
MouseNet v2	Integrated network	17 714	788 080
Component networks			
MM-CX	By co-expression of mouse genes	14 087	180 037
MM-GN	By gene neighborhood of two bacterial orthologs of mouse genes in prokaryotic genomes	4608	157 769
MM-PG	By phylogenetic profile similarity across species	6376	231 833
MM-LC	By literature curated mouse PPIs downloaded from iRefIndex v14.0	5289	11 678
DM-CX	By co-expression of fly (*D. melanogaster*) orthologs	4047	31 951
DM-LC	By literature curated fly orthologous PPIs	1316	3240
HS-HT	By high-throughput human orthologous PPIs	4558	16 487
HS-LC	By literature curated human orthologous PPIs	13 676	163 754
SC-CC	By co-citation of yeast (*S. cerevisiae*) orthologs in Pubmed articles	3094	43 148
SC-CX	By co-expression of yeast orthologs	1996	40 804
SC-GT	By genetic interactions of yeast orthologs	1692	12 526
SC-HT	By high-throughput yeast orthologous PPIs	2304	41 735
SC-LC	By literature curated yeast orthologous PPIs	2553	25 891

## ASSESSMENT AND APPLICATIONS

### Network assessment

We used multiple tests of functional and phenotypic predictive ability to assess the performance of MouseNet v2, and its improvement over prior mouse gene networks. There are several publically-available gene network models for mouse genes derived by integrating genomics data, including STRING v10 (15), Funcoup v3 (16) and Princeton mouseNET (17). A fair comparison of these networks requires a validation data set that is independent of the training and input data for all the networks, which are predominantly trained using GOBP or KEGG pathway database (18), but can also directly incorporate gene pairs that share mammalian phenotype (MP) annotations (19), as for MouseNet v1. Thus, in order to robustly assess the networks, we purposely avoided validation data sets based on mouse pathway or phenotype annotations which are biased toward subsets of the networks being compared.

Given the considerations above, we assessed the networks using the following four sets of gold standard reference gene pairs: (i) gene pairs that belong to the same protein complexes annotated by the CORUM database (20), (ii) protein–protein interactions from the Reactome database (21), (iii) gene pairs linked to the same human diseases as annotated by the Online Mendelian Inheritance in Man (OMIM) database (22) and (iv) gene pairs associated with the same diseases according to the genome-wide association study (GWAS) catalog database (23). Although we cannot completely exclude circularity between these validation data sets and input or training data used for the networks, a network that consistently shows high performance across the validation sets can be considered as performing well.

**Figure 1.**
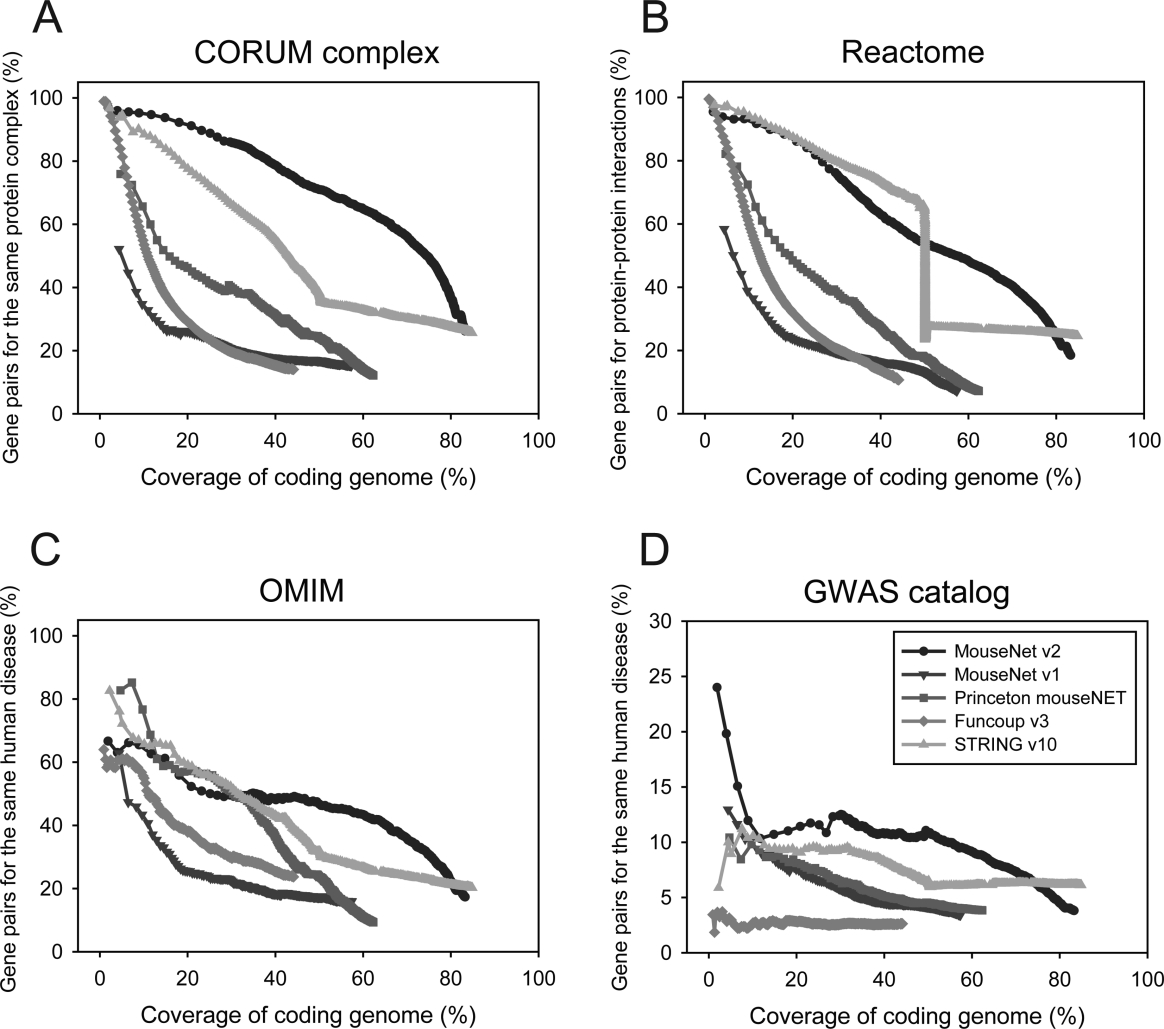
Performance assessment of publicly available functional gene networks for the laboratory mouse. The results of assessment based on precision of network gene pairs for the same protein complexes by CORUM database (**A**), for protein–protein interactions by Reactome database (**B**), for the same human diseases by OMIM database (**C**) or GWAS catalog (**D**) for the given coverage of mouse coding genome suggests that MouseNet v2 generally performs better than other mouse functional gene networks including MouseNet v1.

We observed substantially higher performance for MouseNet v2 than for the other networks for retrieval of gene pairs for the same CORUM protein complexes (Figure 1A). For assessment using Reactome protein-protein interactions, both MouseNet v2 and STRING v10 showed the top performance, although the STRING performance curve declined beyond ∼50% of genome coverage (Figure 1B). Mouse gene networks can also be used to study human disease genes by considering human-mouse orthologs. We tested the retrieval rate of gene pairs for the same human OMIM diseases, and found that three networks, Princeton mouseNET, STRING v10, MouseNet v2, all performed well, with MouseNet v2 showing slightly lower precision for the top several thousand links (Figure 1C). Next, we assessed networks for their ability to identify genes linked to the same human diseases in the GWAS catalog. There are two GWAS disease gene sets: genes ‘reported’ by authors and genes ‘mapped’ in the GWAS catalog database. We generated a validation set based on the reported gene set. To avoid misleading conclusions due to a few dominant GWAS phenotypes, we excluded ‘height’ and ‘obesity’, which annotate 322 genes and 559 genes, respectively. Notably, we observed superior performance for MouseNet v2 over the other networks in retrieving gene pairs associated by GWAS with the same diseases (Figure 1D). The similar analysis using a validation set based on the sets of mapped genes by the GWAS catalog database also supported superiority of MouseNet v2 over all other networks in the comparison (data not shown). Taken together, we conclude that MouseNet v2 represents a significant improvement over prior networks for correctly linking genes to pathways and, by orthology, to diseases.

For each of the validation data sets, MouseNet v2 shows substantially higher precision than MouseNet v1 across the full range of genome coverage. MouseNet v2 incorporates numerous updates to both data sources and network inference methods, as summarized in Supplementary Table S1, all of which have likely contributed to the improved performance. Firstly, there was dramatic growth in the amount of input data available for network inferences. The previous network was developed within the context of the MouseFunc competition (3), which artificially limited both training and input data. In contrast, MouseNet v2 could draw from a wider range of publicly available data, notably the large amount of mouse gene expression data available from GEO and many more sequenced genomes for comparative genomics network inference. Moreover, many evolutionary conserved functional couplings transferred from human, fly (7), and yeast (24), could be incorporated into the new mouse network. Second, we improved the algorithms for inferring networks from genomic context information, by integrating distance- and probability-based measures to improve the gene neighborhood method (11), and by incorporating within-domain co-inheritance analyses to improve the phylogenetic profiling method (7).

### Network-assisted hypothesis generation

**Figure 2.**
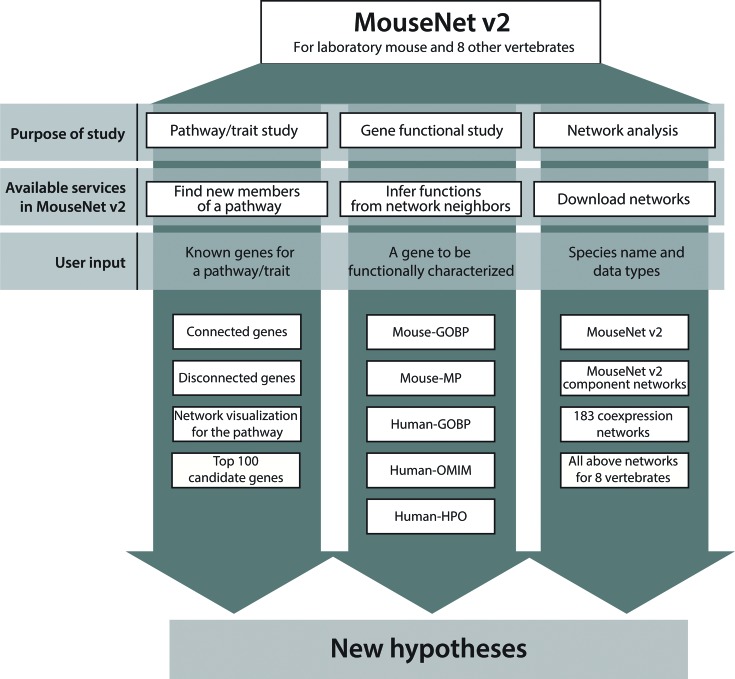
Overview of network-assisted research tools in the MouseNet v2 database. MouseNet v2 provides two network search options, one is for finding new member genes of a pathway/trait and the other is for inferring functions for a query gene from its network neighbors. In addition, MouseNet v2 provides all network information including eight other vertebrate species, enabling various network analysis for mouse and other vertebrates with the integrated networks, component networks for distinct types of data, and co-expression networks for different biological contexts.

The MouseNet v2 database serves as a research platform for generating hypotheses about gene function. The options for hypothesis generation in MouseNet v2 are summarized in Figure 2.

#### Find new members of a pathway/trait (pathway-centric network search)

**Figure 3.**
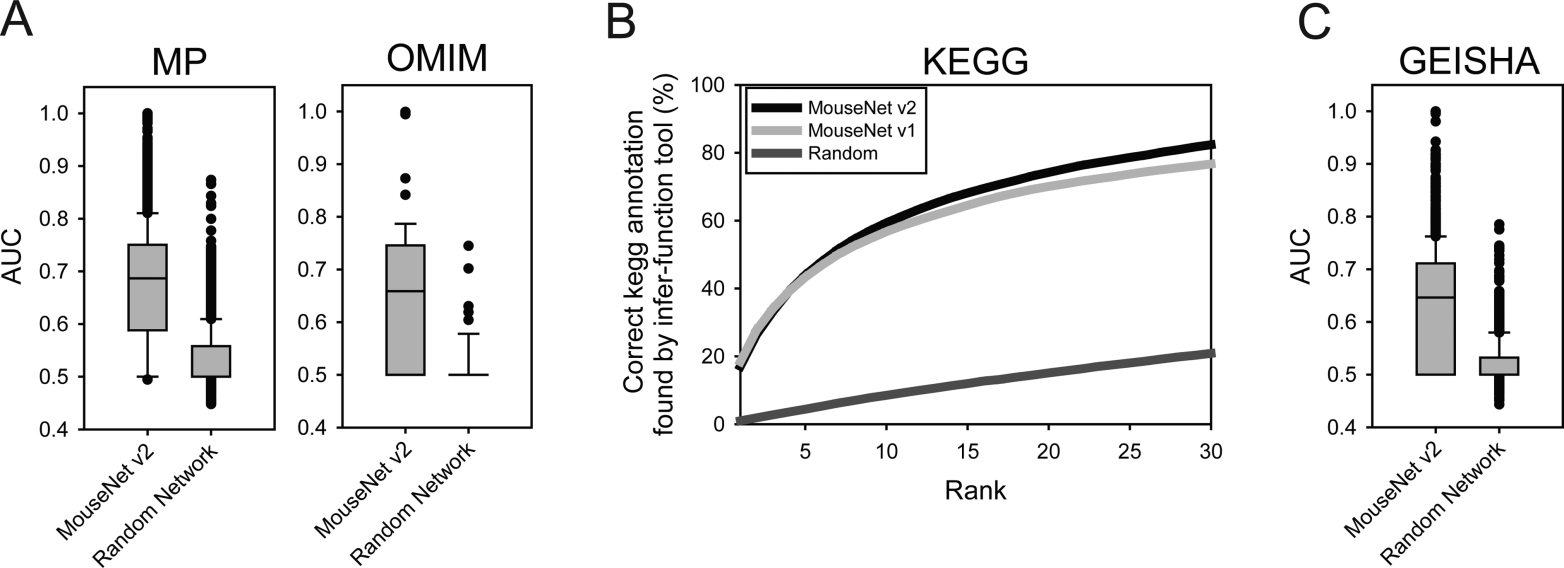
Validating predictions by MouseNet v2. (**A**) Validation of predictions for new members for a pathways using mouse phenotype and human disease database. If known genes for a MP or OMIM term are well connected to each other in the network, network-based prediction would predict new genes for the same MP or OMIM term. The interconnectivity among the known genes for a phenotype was analyzed by ROC curve which was then summarized into AUC. MouseNet v2 shows substantially higher distribution of AUCs for 5424 MP terms and 56 mouse OMIM terms compared with randomized networks. (**B**) Validation of predictions for new functional concepts for a query gene. We have run the prediction for KEGG pathway terms, and count the number of mouse genes whose correct KEGG annotation was retrieved within top N ranks. For example, known KEGG annotations for ∼60% of tested mouse genes was retrieved within top 10 predictions by MouseNet v2 ‘Infer functions from network neighbors’ option, whereas only ∼5% was so by randomized networks. (**C**) Validation of predictions for new member genes for a pathways in chicken using spatiotemporal expression data of chicken genes based on GEISHA database. MouseNet v2 shows substantially higher distribution of AUCs for 1749 spatiotemporal expression sets by GEISHA database compared with randomized networks.

MouseNet v2 can prioritize candidate genes for a pathway/trait of interest. The study of complex traits such as polygenic diseases can be facilitated by network analysis, because genes for a phenotype or disease tend to be functionally associated (25). Thus, we implemented pathway-centric network search algorithm, in which known genes for a pathway/trait provided by the user *guide* the search for new candidates in the network. If a set of genes known for a pathway term are already interconnected in a functional network, new genes that are connected to the known genes are likely to be involved in the same pathway. To test whether MouseNet v2 connects known genes for the same mouse or human phenotype, all mouse genes were ranked by edge-weighted connectivity to the known phenotype genes based on MP or mouse OMIM annotation, and then the retrieval rate for the known phenotype-linked genes was measured by receiver operating characteristic (ROC) analysis and summarized by the area under the ROC curve (AUC). We observed significantly higher AUC scores for gene sets for 5424 MP terms and 56 mouse OMIM terms (with at least four member genes) for MouseNet v2, as compared to random networks (*P*-value < 1e−16 and 1e−7 for MP and OMIM, respectively; Wilcoxon signed rank sum test) (Figure 3A). These results suggest that MouseNet v2 can facilitate discovery of novel genes for many mammalian phenotypes, as well provide insights into human diseases.

We also tested feasibility of identification of novel genes for a pathway by performing the pathway-centric network search. We submitted 41 mouse genes annotated by a GOBP term, innate immune response, and found that they are highly predictive by MouseNet v2, as indicated by high AUC score (AUC = 0.77). The majority of new candidate genes turned out to be ones annotated by closely related GOBP terms such as cellular response to lipopolysaccharide, activation of innate immune response, dendritic cell proliferation, and response to virus. Notably, a top candidate gene, Parp12 (rank 9) was not annotated by GOBP, but recently reported as an interferon induced gene with a potential role in cellular defenses against viral infections (26).

#### Infer functions from network neighbors (gene-centric network search)

The original aim of the MouseFunc competition (3) was to expand functional annotation of mouse genes. Although significant improvements in functional annotation have been achieved over the past several years, there are still ∼61% of mouse genes (12 186 genes) with no GOBP annotation based on direct experimental evidence. Currently, ∼24% of the genes (4869 genes) are completely unannotated by any GOBP evidence including computational methods. Thus, a significant portion of the mouse genome remains to be functionally characterized.

With functional networks, candidate functions can be inferred by searching for enriched functions among network neighbors of a query gene. Interestingly, MouseNet v2 contains 3,852 of the 4,869 completely uncharacterized genes and 10 063 of the 12 186 genes with no reliable functional annotation, and thus provides new opportunities for functional annotation of the majority of uncharacterized genes, capable of suggesting candidate functions for targeted validation. A step-by-step guide on how to prioritize the candidate annotations for a gene using the gene-centric search option is available from the manual page (http://www.inetbio.org/mousenet/tutorial.php). We found that 2770 of the 4869 completely unannotated genes were predicted by any GOBP term. The MouseNet v2 database serves predictions for not only functions but also phenotypes/diseases as cataloged in six annotation databases: (i) mouse GOBP, (ii) mouse KEGG, (iii) MP, (iv) human GOBP, (v) human phenotype ontology (HPO) (27) and (vi) OMIM. To test performance of the network search options for inferring functions, we measured the retrieval rate of correct KEGG terms within top *n* candidates, and found that MouseNet v2 performs better than MouseNet v1 (Figure 3B).

To demonstrate feasibility of functional annotation of mouse genes using MouseNet v2, we performed gene-centric network search for several mouse genes that were not annotated by GOBP and validated the predicted GOBP terms by the literature. For example, three completely unannotated genes, Adam4, Scgn, and Synpo2 were predicted for GOBP terms of ‘binding of sperm to zona pellucida’ (rank 1), ‘retinal bipolar neuron differentiation’ (rank 1), and ‘muscle contraction’ (rank2), respectively, and all of these predictions were validated by experimental results from the literature (28–30).

#### Network information for eight other model vertebrates

Besides the laboratory mouse, several other model vertebrates are often used in various research areas, notably rat (*R. norvegicus*), chimpanzee (*P. troglodytes*), Rhesus macaque (*R. macaque*), dog (*C. lupus familiaris*), cattle (*B. taurus*), chicken (*G. gallus domesticus*), western clawed frog (*X. tropicalis*), zebrafish (*D. rerio*). The MGI database (5) provides mouse orthology information for these vertebrate models, so we expanded the MouseNet v2 server to allow network searches for genes of these model vertebrates. For example, MouseNet v2 server provides an example query of 23 zebrafish genes involved in heart morphogenesis (GO:0003007). A pathway-centric network search, using as a query the 23 zebrafish genes associated (via orthology) with human cardiovascular diseases, returns strong candidate genes including gata4 (rank 1), smarca4a (rank 2), gata6 (rank 3), gata3 (rank 6), scn4aa (rank 10), cacna1da (rank 15), and csrp3 (rank 16). In addition, we performed systematic validation of the predictive power of MouseNet v2 for 1749 sets of chicken genes sharing spatiotemporal mRNA expression patterns, as annotated by the Gallus Expression in Situ Hybridization Analysis (GEISHA) database (31). We found that chicken genes with similar tissue/organ and developmental stage expression patterns are significantly interconnected in MouseNet v2 (Figure 3C, *P-value* < 1e−16 by Wilcoxon signed rank sum test), supporting the application of MouseNet v2 to the study of other vertebrate models. Network data for all eight vertebrates are available from the MouseNet v2 database.

## CONCLUSIONS

In this study, we present an improved functional gene network for the laboratory mouse, MouseNet v2, and demonstrate its improved performance for the study of laboratory mouse gene functions. We confirmed that MouseNet v2 shows good predictive power for genes linked to specific mammalian phenotypes and human diseases, neither of which was explicitly incorporated into the network construction. Thus, a functional interaction map of mouse genes reveals associations between genes and complex traits in the laboratory mouse, as well as humans. Tests of MouseNet v2 on chicken gene mRNA expression patterns suggest that it generally useful for the study of other vertebrate model organisms as well. All of the functional gene networks are released for free and can be searched using the MouseNet v2 web server, which offers a useful resource for mouse, human and other vertebrate genetics.

## SUPPLEMENTARY DATA

Supplementary Data are available at NAR Online.
